# Differences in vertebral morphology around the apical vertebrae between neuromuscular scoliosis and idiopathic scoliosis in skeletally immature patients: a three-dimensional morphometric analysis

**DOI:** 10.1186/s12891-017-1801-0

**Published:** 2017-11-16

**Authors:** Takahiro Makino, Yusuke Sakai, Masafumi Kashii, Shota Takenaka, Kazuomi Sugamoto, Hideki Yoshikawa, Takashi Kaito

**Affiliations:** 1 Department of Orthopaedic Surgery, Osaka University Graduate School of Medicine, 2-2, Yamadaoka, Suita, Osaka, 565-0871 Japan; 2 Department of Orthopedic Biomaterial Science, Osaka University Graduate School of Medicine, 2-2, Yamadaoka, Suita, Osaka, 565-0871 Japan

**Keywords:** Wedging, Vertebral body, Asymmetry, Idiopathic scoliosis, Neuromuscular scoliosis

## Abstract

**Background:**

Recent morphological analyses of vertebrae in patients with scoliosis have revealed three-dimensional (3D) deformities in the vertebral bodies. However, it remains controversial whether these deformities are secondary changes caused by asymmetrical vertebral loading or primary changes caused by aberrant asymmetrical vertebral growth. Furthermore, the difference in vertebral morphology between scoliosis with different pathogeneses remains unclear. This study was aimed to investigate the difference in the coronal asymmetry of vertebral bodies between neuromuscular scoliosis (NS) in Duchenne muscular dystrophy (DMD) and idiopathic scoliosis (IS) using in vivo 3D analysis.

**Methods:**

Twelve male skeletally immature patients with NS in DMD and 13 female skeletally immature patients with IS who underwent corrective fusion at our institution were included retrospectively. 3D bone models of the apical and adjacent upper and lower vertebrae in the major curve in the NS patients and in the main and compensatory curves in the IS patients were constructed using an image processing workstation. The heights of the concave and convex sides of the vertebral bodies were measured at the anterior, middle, and posterior and the concave-to-convex vertebral height ratios (VHR) were calculated.

**Results:**

The mean VHRs (anterior/middle/posterior) for the main curve for IS (0.897 ± 0.072/0.832 ± 0.086/0.883 ± 0.059) were significantly smaller than those for NS (0.970 ± 0.048/0.934 ± 0.081/0.958 ± 0.043) in all three parts (*p* < 0.001). Those of the compensatory curve in IS (0.968 ± 0.045/0.942 ± 0.067/0.967 ± 0.046) did not differ significantly from the NS values in any part.

**Conclusions:**

When compared to the wedging of the vertebral bodies around apical vertebrae in the major curve in NS, which was caused by asymmetric loading, the wedge deformities in both the main and compensatory curves in IS were more severe than would be expected. Our results indicated that morphometric characteristics of vertebral bodies differed according to the pathogenesis of scoliosis and that the pathology of the wedging of vertebral bodies in IS could not be a result only of asymmetric loading to the vertebral bodies.

## Background

Recent morphological analyses of vertebrae in patients with idiopathic scoliosis (IS) have revealed three-dimensional (3D) deformities in the vertebral bodies such as wedging and torsion [1–4]. However, it remains controversial whether these deformities are secondary changes caused by asymmetrical vertebral loading or primary changes caused by aberrant asymmetrical vertebral growth [5]. Furthermore, how the vertebral morphology differs between scoliosis with different pathogeneses remains unclear. We hypothesized that morphometric characteristics of the vertebral bodies differed according to the pathogenesis of scoliosis.

Duchenne muscular dystrophy (DMD) is one of the causes of neuromuscular scoliosis (NS). The muscle weakness and pelvic imbalance that arise in the natural history of DMD induce the development of secondary scoliosis [6, 7]. The progression of scoliosis in DMD involves the whole thoracic and lumbar spine, and so the shape is often described as “C-type” [6]. Because the scoliotic change in patients with DMD is not caused by primary vertebral wedging, establishing the differences in the morphometric characteristics of vertebral bodies between IS and NS in DMD could help to clarify the pathology of vertebral deformities in patients with IS.

Scoliosis is a 3D deformity with vertebral rotation in an axial plane that increases with curve progression. Because of this, morphological analyses of vertebrae by conventional two-dimensional (2D) radiographs in scoliotic patients can be misleading because these cannot show true frontal (coronal) or lateral (sagittal) views of each vertebra [8]. We previously established an in vivo method using computed tomography (CT) scans for the 3D morphological analysis of vertebral bodies in patients with scoliosis [9]. The purpose of the present study was to investigate the difference in the wedging of vertebral bodies between NS in DMD and IS in skeletally immature patients using this in vivo 3D analysis.

## Methods

### Subjects

This study was a retrospective review of a radiological database of patients with IS and NS in DMD who underwent corrective surgery, and was approved by the Research Ethics Committee of Osaka University Hospital (no. 15098–2).

Twelve consecutive patients with NS in DMD who underwent corrective spinal fusion surgery between 2010 and 2015 were included in this study (the NS Group). All patients in the NS Group were male. The mean age at the time of the surgery was 12.9 years (range, 12–15 years) and the mean Risser grade was 0.6 (range, 0–3). Thirteen consecutive skeletally immature patients (Risser grades ≤3) with IS who underwent corrective spinal fusion surgery between 2008 and 2015 were also included (the IS Group). All patients in the IS Group were female. Their mean age at the time of the surgery was 12.1 years (range, 10–14 years) and the mean Risser grade was 1.7 (range, 0–3).

The periods between the loss of ambulation and surgery and the use of steroids in the NS Group were obtained from the medical charts.

### Radiographic assessments

From preoperative full-length posteroanterior radiographs obtained in the sitting (NS Group) or standing (IS Group) position, the apical vertebrae were determined for the major curve in the NS Group and for the main thoracic (MT) and thoracolumbar/lumbar (TL/L) curves in the IS Group. Cobb angles of the major curve in the NS Group and of the MT and TL/L curves in the IS Group were digitally measured on a flat-panel monitor at our hospital using built-in imaging software (Centricity WebDX; GE Healthcare Japan, Tokyo, Japan). These Cobb angles were also measured from preoperative supine bending posteroanterior radiographs. The flexibility of each curve was evaluated and the type of scoliosis was classified as a structural or non-structural curve according to the Lenke classification [10]. In the IS Group, the structural curve was defined as the main curve and the non-structural curve as the compensatory curve; if both curves were flexible (non-structural), the greater curve when in the standing position was defined as the main curve. The flexibility index (FI, %) of each curve was calculated from the following formula:

$$\begin{aligned} \textbf{FI}(\%) = & \left(\left[\textit{Preoperative\, standing\, or\, sitting\, Cobb\, angle}\right]-\right. \\
& \, \, \left.\left[\textit{Preoperative\, supine\, bending\, Cobb\, angle}\right]\right)/ \\
& \left(\textit{Preoperative\, standing\, or\, sitting\, Cobb\, angle}\right) \times 100\%.
 \end{aligned} $$

### Computed tomography assessments

**Fig. 1:**
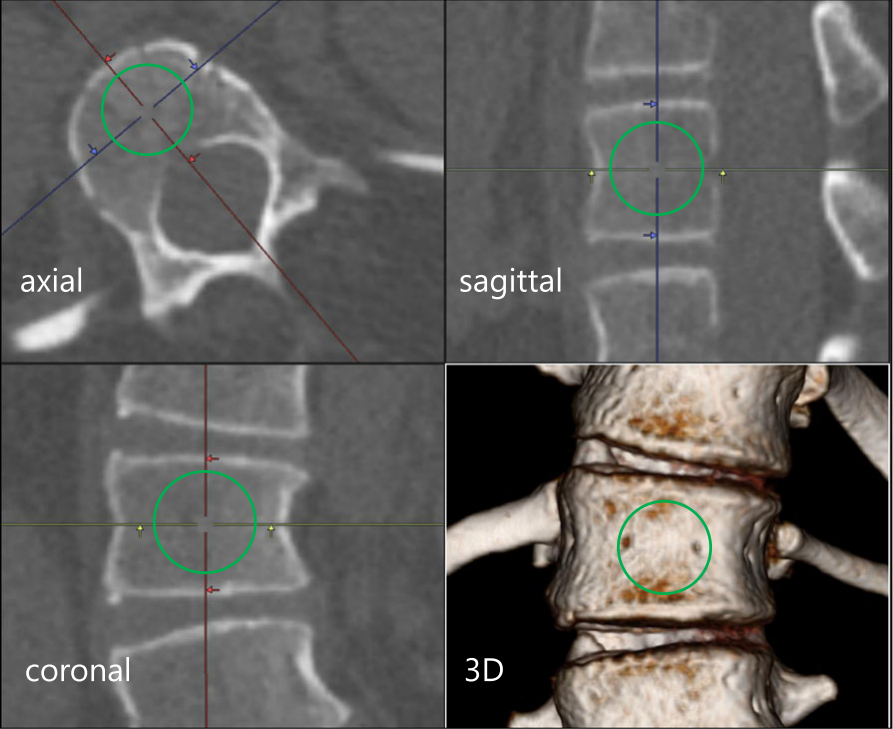
An example of the measurement of the Hounsfield Unit (HU) value for a vertebral body to assess bone mineral density. The largest possible spherical region of interest (ROI, *green line*) that excluded the cortical margin was placed on the vertebral body. The center of the spherical ROI was set to be the center of the vertebral body on the axial, coronal, and sagittal planes. The HU value of the ROI was then calculated automatically

The patients underwent CT scans within 3 months preoperatively using Discovery CT750HD (GE Healthcare Japan, Tokyo, Japan) or Aquilion ONE (Toshiba Medical Systems Corporation, Tochigi, Japan) scanners. The settings used for the scans were a slice thickness of 0.625 mm with the Discovery CT750HD and 0.5 mm with the Aquilion ONE, and a tube voltage of 120 kVp. The tube current was maintained between 50 and 250 mA by an automatic exposure control system. Hounsfield unit (HU) values of the vertebral bodies of the apical vertebrae and the adjacent upper and lower vertebrae were measured from the CT scans to assess the bone mineral density (BMD) by the following method (Fig. 1). First, the largest possible spherical region of interest (ROI) that excluded the cortical margin was placed on each vertebral body, with its center set to be the center of the vertebral body on the axial, coronal, and sagittal planes. The HU value of the ROI was then calculated automatically by built-in imaging software (Synapse Vincent; Fujifilm Holdings Corporation, Tokyo, Japan). The mean HU values for each curve were calculated.

### Segmentation and creation of a 3D bone surface model and measurement of vertebral height

**Fig. 2:**
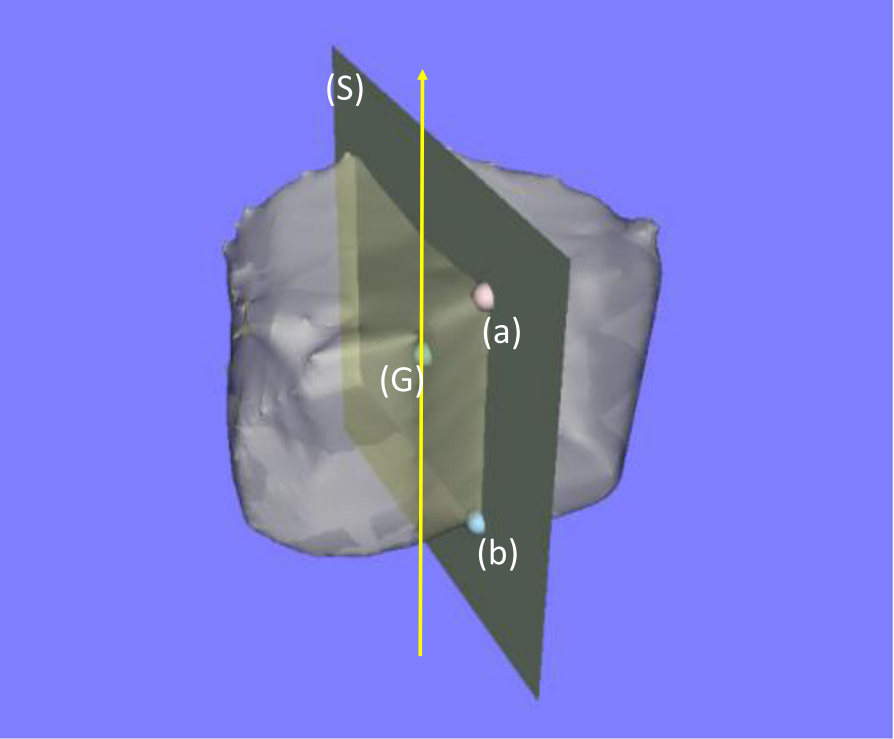
The vertical axis (arrow) was defined as the line passing through the center of gravity (**G**) parallel to the line connecting the anterior edges of the vertebral foramen on the upper and lower vertebral end plates (**a**, **b**). The sagittal plane (**S**) contained the center of gravity (**G**) and the anterior edges of the vertebral foramen on the upper and lower end plates (**a**, **b**)

**Fig. 3:**
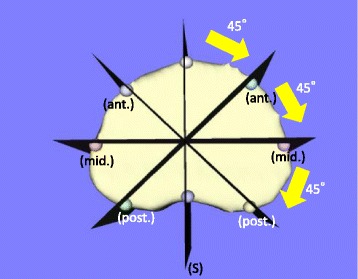
The sagittal plane (S) was rotated about the vertical axis at 45° intervals. The two anterior intersection points (ant.) between the rotated sagittal planes and the lower endplate were used for assessing the anterior part of the vertebral body, the middle two intersection points (mid.) for assessing the middle part, and the posterior two intersection points (post.) for assessing the posterior part

**Fig. 4:**
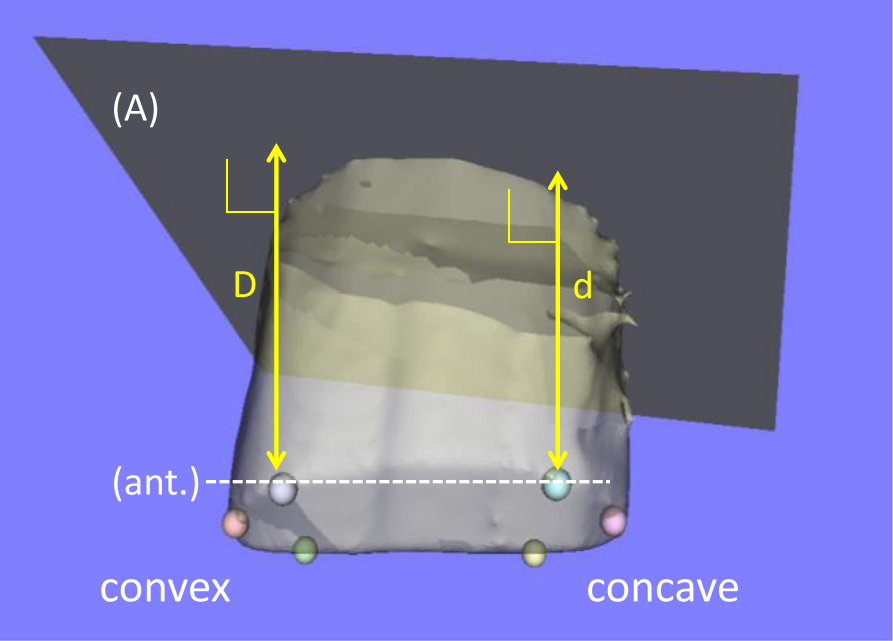
Calculation of the vertebral height ratio (VHR). A plane approximating the upper endplate (A) was produced automatically by custom-made software (Orthopedic Viewer, Osaka University). The vertebral heights (D and d) were defined as the distances between the two intersection points and the plane. VHR was defined as the ratio of the vertebral height of the concave side to that of the convex side (e.g., the VHR at the anterior = d/D)

A 3D bone surface model of each vertebra was segmented and created semi-automatically by our previously reported method using a 3D image processing workstation (Synapse Vincent; Fujifilm Holdings Corporation, Tokyo, Japan) [9]. From these, 3D models of the apical vertebrae and the adjacent upper and lower vertebrae in the major curve in the NS Group and in both MT and TL/L curves in the IS Group were constructed, resulting in 36 3D models of vertebrae in the NS Group and 78 in the IS Group. The vertebral bodies were then extracted semi-automatically from the 3D models of the vertebrae by removing the posterior elements at the transitions between the vertebral bodies and pedicles. From the 3D models, vertebral height was measured semi-automatically using the original digital viewer (Orthopedic Viewer; Osaka University, Osaka, Japan), as described in our previous report in detail (Figs. 2, 3 and 4) [9]. Then, vertebral height ratio (VHR: the ratio of the vertebral height of the concave side to that of the convex side) was calculated as the index of wedge deformity of the vertebral bodies in the coronal plane. VHR was assessed at the anterior, middle, and posterior of each vertebral body. A value of VHR close to 1.0 indicated that the upper and lower endplates of the vertebral body in the frontal plane were nearly parallel.

### Statistical analysis

The statistical analysis was performed using IBM SPSS Statistics Version 22 (IBM, Armonk, NY, USA). The Mann–Whitney U-test was used to compare variables. Differences in age between the NS and IS Groups were considered statistically significant at *p* < 0.05. Statistical significance for the other variables related to the curves (the major curve in the NS Group and the main curve and compensatory curves in the IS Group) was set as *p* < 0.017 after applying the Bonferroni correction.

## Results

**Fig. 5:**
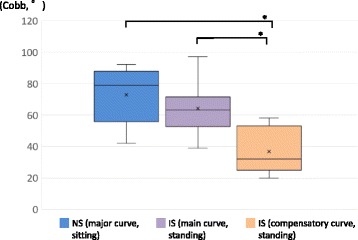
Comparisons of the Cobb angles of the major curve in the neuromuscular scoliosis (NS) Group (sitting) and the main and compensatory curves in the idiopathic scoliosis (IS) Group (standing). In each plot, × indicates the mean value, the horizontal line indicates the median, the box shows the interquartile range, and the vertical lines indicate the overall range. *p < 0.001

**Fig. 6:**
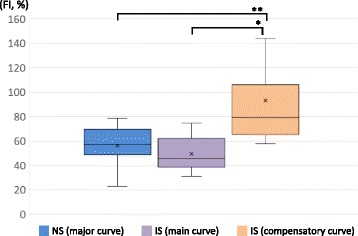
Comparisons of the Flexibility index (FI) of the major curve in the neuromuscular scoliosis (NS) Group and the main and compensatory curves in the idiopathic scoliosis (IS) Group. In each plot, × indicates the mean value, the horizontal line indicates the median, the box shows the interquartile range, and the vertical lines indicate the overall range. *p < 0.001, **p = 0.005

**Fig. 7:**
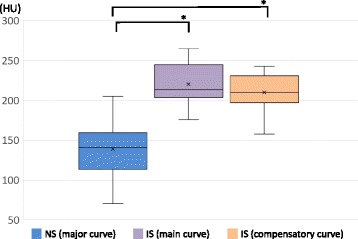
Comparisons of the Hounsfield Unit (HU) of the major curve in the neuromuscular scoliosis (NS) Group and the main and compensatory curves in the idiopathic scoliosis (IS) Group. In each plot, × indicates the mean value, the horizontal line indicates the median, the box shows the interquartile range, and the vertical lines indicate the overall range. *p < 0.001

The demographic and radiographic data of each group are shown Tables 1 and 2. The mean age did not differ significantly between the Groups (*p* = 0.20). All patients in the NS Group exhibited “C-type” coronal curves. In the IS Group, three patients exhibited Lenke type 1 curves, seven patients, type 2, and three patients, type 5. There was no significant difference between the Cobb angles of the major curve in the NS Group in the sitting position and the main curve in the IS Group in the standing position (*p* = 0.26); however, the Cobb angle of the compensatory curve in the IS Group was significantly smaller than that of the major curve in the NS Group (*p* < 0.001) (Fig. 5). In the IS Group, the Cobb angle of the main curve was greater than that of the compensatory curve (*p* < 0.001) (Fig. 5). The FI of the major curve in the NS Group did not differ significantly from that of the main curve in the IS Group (*p* = 0.19); however, the FI of the compensatory curve in the IS Group was significantly greater than that of the major curve in the NS Group (*p* = 0.005) (Fig. 6). In the IS Group, the FI of the main curve was smaller than that of the compensatory curve (*p* < 0.001) (Fig. 6). The HU values of the major curve in the NS Group were smaller than those of the main and compensatory curves in the IS Group (*p* < 0.001) (Fig. 7). In the IS Group, the HU values did not differ between the main curve and the compensatory curve (*p* = 0.34) (Fig. 7).

**Table 1:** Demographic and radiographic data for the neuromuscular scoliosis group (*n* = 12)

Characteristic	
Age (years)	12.9 ± 1.1
Risser grade (0:1:2:3)	9:0:2:1
Period between loss of ambulation and surgery (month)	45.0 ± 18.4
Steroid use (yes: no)	3: 9
Configuration of major curve	
Apical vertebra (no. of patients)	T12, 1; L1, 4; L2, 4; L3, 3
Cobb angle (°) in the sitting position	73.0 ± 16.8
Cobb angle (°) in the supine bending position	31.8 ± 13.9
Flexibility index (%)	56.4 ± 16.1
Hounsfield unit (HU)	139.0 ± 33.3

Values are expressed as means ± standard deviations

**Table 2:** Demographic and radiographic data for the idiopathic scoliosis group (*n* = 13)

Characteristic	
Age (years)	12.1 ± 1.3
Risser grade (0:1:2:3)	3:2:4:4
Lenke classification (no. of patients)	Type 1, 3; Type 2, 7; Type 5, 3
Configuration of main and compensatory curves	
**Main curve**	
Apical vertebra (no. of patients)	T8, 3; T9, 5; T10, 2; T12, 2; L1, 1
Cobb angle (°) in the standing position	64.2 ± 16.1
Cobb angle (°) in the supine bending position	31.5 ± 8.8
Flexibility index (%)	49.5 ± 13.3
Hounsfield unit (HU)	220.1 ± 25.2
**Compensatory curve**	
Apical vertebra (no. of patients)	T3, 1; T6, 1; T7, 1; L2, 2; L3, 5; L4, 3
Cobb angle (°) in the standing position	36.8 ± 13.3
Cobb angle (°) in the supine bending position	4.5 ± 10.8
Flexibility index (%)	93.0 ± 37.6
Hounsfield unit (HU)	209.5 ± 23.5

Values are expressed as means ± standard deviations

**Fig. 8:**
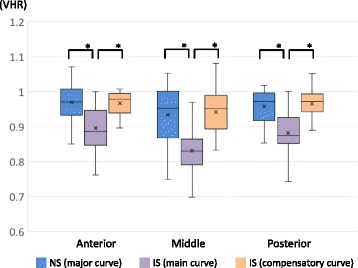
Comparisons of the vertebral height ratios (VHR) of the major curve in the neuromuscular scoliosis (NS) group and the main and compensatory curves in the idiopathic scoliosis (IS) group measured at the anterior, middle, and posterior parts of the vertebral bodies. The further the value of a VHR diverges from 1.0, the greater the severity of the wedge deformity of the vertebral body in the frontal plane. In each plot, × indicates the mean value, the horizontal line indicates the median, the box shows the interquartile range, and the vertical lines indicate the overall range. *p < 0.001

The VHRs for the major curve in the NS Group and the main and compensatory curves in the IS Group are presented in Table 3. The VHR for the main curve in the IS Group was significantly smaller (further from 1.0) than that for the major curve in the NS Group and the compensatory curve in the IS Group at the anterior, middle, and posterior of the vertebral bodies (all *p* < 0.001) (Fig. 8). In contrast, there was no significant difference in the VHRs for the compensatory curve in the IS Group and the major curve in the NS Group for any part of the vertebral bodies (anterior, *p* = 0.54; middle, *p* = 0.87; posterior, *p* = 0.64) (Fig. 8).

**Table 3:** Vertebral height ratios for the major curve in the neuromuscular scoliosis (NS) group and the main and compensatory curves in the idiopathic scoliosis (IS) group

Characteristic	Anterior	Middle	Posterior
NS Group			
Major curve	0.970 ± 0.048	0.934 ± 0.081	0.958 ± 0.043
IS Group			
Main curve	0.897 ± 0.072	0.832 ± 0.086	0.883 ± 0.059
Compensatory curve	0.968 ± 0.045	0.942 ± 0.067	0.967 ± 0.046

Values are expressed as means ± standard deviations

## Discussion

This study revealed that the morphology of vertebral bodies differed according to the pathogenesis of scoliosis, in accordance with our hypothesis. First, the wedging of vertebral bodies in the main curve in the IS Group was more severe than that in the major curve in the NS Group across the whole vertebral body (anterior, middle, and posterior), although the severity and flexibility of scoliosis did not differ between the curves. Second, the wedge deformity in the compensatory curve in the IS Group was similar to that in the major curve in the NS Group across the whole vertebral body, although the curve of the compensatory curve in the IS Group was less severe and more flexible than that of the major curve in the NS Group. To the best of our knowledge, this is the first study to compare vertebral morphology between patients with NS in DMD and patients with IS.

It has been well known that vertebral wedge deformities can occur in patients with scoliosis [1–4, 11], and these deformities can be primarily obvious in the frontal plane [1, 12]. Several authors have shown that the deformities became more severe according to increase in Cobb angle and that this deformities was greatest in the apical region by 3D morphometric analyses [1, 11]. We therefore, investigated the morphology of vertebral bodies in the frontal plane around apical lesions. We showed in our previous report that intra-class correlation coefficients for intra-observer and inter-observer reliabilities for the calculation of vertebral heights were 0.996 (95% confidence interval, 0.994–0.997) and 0.990 (0.986–0.993) [9].

It is well established that mechanical loading influences the longitudinal growth of the long bones and vertebrae. This phenomenon is known as the Hueter–Volkmann Law, which explains that growth is retarded by increased mechanical compression and accelerated by decreased loading [13]. Stokes et al. [14] revealed that a compression force could suppress the longitudinal growth of vertebrae and a distraction force could accelerate it by using a rat-tail model. Meir et al. [15, 16] demonstrated that the loading in the intervertebral disc in the concave annulus was greater than that in the convex annulus in patients with scoliosis in vivo. The vicious cycle of asymmetrical loading to the intervertebral disc and vertebral wedge deformities can continue in patients with scoliosis [5, 14]. The effect of asymmetrical loading and severity of vertebral wedge deformities can be influenced by the BMD of the vertebral bodies. However, it is difficult to measure BMD of each vertebral body from thoracic to lumbar spine by conventional dual-energy X-ray absorptiometry measurements. Thus, we measured HU values of the vertebral bodies directly from the CT scans because the HU values reportedly correlate with BMD [17].

It has been recognized in the natural history of patients with NS in DMD that the loss of function for maintaining their posture due to muscle weakness and pelvic imbalance results in the development of their scoliosis [6, 7]. Thus, scoliosis in patients with NS in DMD does not originate from the wedging of vertebral bodies; these wedge changes of the vertebral bodies are secondary to asymmetric loading. The progression of scoliosis in patients with NS in DMD reportedly begins after the loss of ambulation [18]. In the present study, the mean period between loss of ambulation and surgery was about 45 months. Thus, our present data for the NS Group represent the natural course of the wedging of vertebral bodies induced by asymmetric loading over several years.

In comparison to this wedging of vertebral bodies in the NS Group, the wedging in the main curve in the IS Group was more severe, although the severity and flexibility of the curves were similar between both types of patient. Furthermore, the wedge deformity of vertebral bodies was similar between the compensatory curve in the IS Group and the major curve in the NS Group despite there being less severity and greater flexibility in the compensatory curve in the IS Group. The wedge deformities in both the main and compensatory curves in the IS Group were more severe than would be expected, because the BMD of vertebral bodies in the NS group was lower than in the IS group and thus the wedging of vertebral bodies was more likely to occur in the NS group only from the perspective of bone quality. These discrepancies in the progression of asymmetry in the vertebral bodies could relate to the difference between NS in DMD and IS in the progression of scoliosis. The scoliosis in NS often progresses rapidly [6, 7], so the asymmetric deformity secondary to asymmetric loading by scoliosis may not be as severe in patients with NS.

The primary factors that affect the vertebral morphology in IS could also contribute to the difference in the asymmetric changes of vertebral bodies between NS in DMD and IS. Several candidate susceptibility genes for adolescent IS have been reported since the development of a genome-wide association study [19–23]. *GPR126* knockdown and *BNC2* overexpression in zebrafish have been shown to cause delayed ossification of the developing spine and scoliosis [19, 20]. Growth arrest at the epiphyseal growth plates at the concave side of the apical vertebrae in patients with IS can be induced by the asymmetrical expression of these genes regulating spine ossification.

There were some limitations to the present study. Because of the retrospective nature of our study, it was not clear when the curves of the patients appeared. Furthermore, the male-to-female ratio was different between the two patient groups because DMD is more likely to occur in male and IS in female. These factors could affect the difference between the patients in the wedging of vertebral bodies. However, we think the effect of the difference in sex on vertebral morphology is relatively small in our study, because age and skeletal maturity did not differ between the groups and both the major curve in the NS Group and the main curve in the IS Group had already equally developed.

## Conclusions

In conclusion, when compared to the frontal wedging of the vertebral bodies around apical vertebrae in the major curve in the patients with NS, which was caused by asymmetric loading, the wedge deformities in both the main and compensatory curves in patients with IS were more severe than would be expected. Our results indicated that morphometric characteristics of vertebral bodies differed according to the pathogenesis of scoliosis and that the pathology of the wedging of vertebral bodies in patients with IS could not be a result only of asymmetric loading to the vertebral bodies. With regard to the clinical relevance of our findings, the evaluation of vertebral wedge deformities can be an index to distinguish idiopathic scoliosis and syndromic scoliosis in adolescent patients.

## Abbreviations

2D: Two-dimensional
3D: Three-dimensional
BMD: Bone mineral density
CT: Computed tomography
DMD: Duchenne muscular dystrophy
FI: Flexibility index
HU: Hounsfield unit
IS: Idiopathic scoliosis
MT: Main thoracic
NS: Neuromuscular scoliosis
ROI: Region of interest
TL/L: Thoracolumbar/lumbar
VHR: Vertebral height ratio
